# Epidemiological Risk Factors Associated with High Global Frequency of Inapparent Dengue Virus Infections

**DOI:** 10.3389/fimmu.2014.00280

**Published:** 2014-06-11

**Authors:** Laura Grange, Etienne Simon-Loriere, Anavaj Sakuntabhai, Lionel Gresh, Richard Paul, Eva Harris

**Affiliations:** ^1^Unité de la Génétique Fonctionnelle des Maladies Infectieuses, Institut Pasteur, Paris, France; ^2^URA3012, Centre National de la Recherche Scientifique, Paris, France; ^3^Sustainable Sciences Institute, Managua, Nicaragua; ^4^Division of Infectious Diseases and Vaccinology, School of Public Health, University of California, Berkeley, CA, USA

**Keywords:** dengue, subclinical, inapparent, asymptomatic, infection

## Abstract

Dengue is a major international public health concern, and the number of outbreaks has escalated greatly. Human migration and international trade and travel are constantly introducing new vectors and pathogens into novel geographic areas. Of particular interest is the extent to which dengue virus (DENV) infections are subclinical or inapparent. Not only may such infections contribute to the global spread of DENV by human migration, but also seroprevalence rates in naïve populations may be initially high despite minimal numbers of detectable clinical cases. As the probability of severe disease is increased in secondary infections, populations may thus be primed, with serious public health consequences following introduction of a new serotype. In addition, pre-existing immunity from inapparent infections may affect vaccine uptake, and the ratio of clinically apparent to inapparent infection could affect the interpretation of vaccine trials. We performed a literature search for inapparent DENV infections and provide an analytical review of their frequency and associated risk factors. Inapparent rates were highly variable, but “inapparent” was the major outcome of infection in all prospective studies. Differences in the epidemiological context and type of surveillance account for much of the variability in inapparent infection rates. However, one particular epidemiological pattern was shared by four longitudinal cohort studies: the rate of inapparent DENV infections was positively correlated with the incidence of disease the previous year, strongly supporting an important role for short-term heterotypic immunity in determining the outcome of infection. Primary and secondary infections were equally likely to be inapparent. Knowledge of the extent to which viruses from inapparent infections are transmissible to mosquitoes is urgently needed. Inapparent infections need to be considered for their impact on disease severity, transmission dynamics, and vaccine efficacy and uptake.

## Introduction

Dengue has become a major international public health concern and is the most important arthropod-borne disease of humans (1–4). Any of the four antigenically distinct viruses, or serotypes, designated dengue virus (DENV)-1, DENV-2, DENV-3, and DENV-4, belonging to the *Flavivirus* genus in the family *Flaviviridae*, can cause dengue fever (DF), an acute viral infection characterized by fever, rash, headache, muscle and joint pain, and nausea, as well as more severe forms of the disease. A possible fifth serotype has recently been detected, but its global significance remains to be seen (5). Occasionally, DF progresses to dengue hemorrhagic fever/dengue shock syndrome (DHF/DSS), a potentially life-threatening illness associated with vascular leakage, hemorrhage, and shock. By contrast, it is increasingly recognized that the majority of DENV infections are subclinical, resulting in insufficient discomfort for clinical consultation (6). This reservoir of infection needs to be addressed.

Over the past decade, the number of dengue outbreaks has escalated (4), and the population at risk is increasing yearly. More than 3.5 billion people are at risk of DENV infection. It has recently been estimated that there are 390 million DENV infections every year, of which up to 96 million are symptomatic (7). This prolific increase has been associated with societal changes such as population growth and increasing urbanization, particularly in tropical cities with poor waste and water management, leading to proliferation of the domestic and peridomestic mosquito species that transmit DENV, *Aedes aegypti* and *A. albopictus*. Human migration and international trade and travel are constantly introducing new vectors and pathogens into novel geographic areas (8, 9). In addition, it has been suggested that rising temperatures and global climate change may lead to the expansion of the range of major mosquito vectors into new areas, extension of the transmission season in areas with currently circulating DENV, decrease in extrinsic incubation period, and increase in the mosquito spp. vectorial capacity (10, 11). The potential threat of DENV invasion of continental Europe has recently been illustrated by cases of autochthonous dengue in southern France (12). These cases bear testament to the capacity of local *A. albopictus* mosquito vector to transmit the virus. This Asian tiger mosquito is the major potential vector of DENV in Europe, although the most important vector world-wide, *A. aegypti*, was identified in Madeira Island, Portugal, in October 2005. A major epidemic occurred in Madeira in 2012 (13). Autochthonous transmission of DENV in the United States has also been reported intermittently over the past decade in Texas, Hawaii, and Florida (14, 15).

International travel will ensure importation of virus into non-endemic countries from regions endemic for dengue. Infected individuals may harbor sufficiently high viral loads to infect mosquitoes prior to the onset of symptoms and thereby introduce the virus into the population. Potentially more important is the epidemiological significance of inapparent, subclinical infections. Travelers may import virus without showing overt clinical symptoms and thus will not be detectable either in the airports or once in the country. There is some suggestion that primary (1°) DENV infections can be majoritarily inapparent in certain outbreaks (16), whereas secondary (2°) infections lead to more severe symptoms even when occurring 20 years later (17). In fact, the longer the interval between heterotypic DENV infections, the higher the case fatality rate (18). The public health consequences of such inapparent infections are considerable, because apparently naïve populations may well have been previously exposed to infections, and once hospital cases of dengue are detected, the population as a whole may have already been primed with prior DENV infection.

The frequency of inapparent infections is extremely variable year to year, the risk factors poorly understood, and the terminology not standardized. Subclinical, inapparent, and asymptomatic infections are often used as synonyms, and the use of paucisymptomatic is used to designate a DENV infection with few symptoms. We will use subclinical and inapparent to denote infections with insufficient symptoms to be detected by the research or national surveillance program and/or to incite the infected individual to consult, but for which there is evidence, either by seroconversion or detection of virus, that the individual was infected with DENV. Asymptomatic infections will be used when there are no symptoms at all reported by the infected individual during an active infection, whether inferred by seroconversion or serology.

We review the literature on the extent of inapparent DENV infections, identify associated risk factors, and highlight several important lacunae that need to be addressed to assess the extent of the epidemiological importance of inapparent infections. We combine a PubMed literature search approach with review of articles cited within PubMed hits, plus a review of the classical pre-PubMed dengue literature. The search strategy was dengue + one of the following: inapparent, asymptomtic, subclinical. This search (27 January 2014) yielded 28, 151, and 34 articles, respectively. Acceptance criteria for selection were: (i) definition of symptom classification, (ii) ascertainment of symptoms and recent/concurrent viral infection at an individual level, and (iii) quantification of the number of inapparent infections. Of the retrieved PubMed articles, 33 publications fulfilled the acceptance criteria (19–51). An additional 14 articles cited within the above fulfilled acceptance criteria (52–65). One further recent article was known to the authors (66). A short description of each study is presented in the Supplementary Material and in Table 1. Below, we highlight features pertinent to dengue epidemiology that these studies elucidate.

**Table 1:** **Prospective studies addressing the inapparent rate**.

Place and reference	Virus (years)	Minimum symptom	% Inapparent (*N*)	Incidence rate: disease; infection	Age (years)	Seroprevalence
Puerto Rico (58)	DENV-4 (1982)	Fever	45 (56)	36%; 31%	All	70%
Nicaragua (59)	DENV-1 and DENV-2 (2001–2003)	Fever	91 (106)	8.3–8.5/1000; 6–12%	4–16	91%
Nicaragua (39, 41, 42)	DENV-1 (2004–2005); DENV-2 (2006–2008); DENV-3 (2008–2010)	Fever or WHO	83 (1778)	3.4–43.5; 67–110 (/1000 person-years)	2–14	30% age 2; 90% age 9
Nicaragua (40)	DENV-2 (2006–2007)	Any	42 (12)	0.2–1.4%; 7%	11–67	ND
Peru (52)	DENV-1 and -2 (1999–2001); DENV-3 (2002–2028); DENV-4 (2008–2011)	Fever	93 (3837)	0.5–19; 2–90 (/100 person-years)	All	60% age 2; 90% age 15
Brazil (38)	DENV-2 and DENV-3 (2007–2008)	Fever+ 2 WHO	77 (30)	2%; ND	1–79	56–77%
Thailand (26)	DENV-1–4 (1980–1981)	Fever	87 (103)	0.74%; 5.9%	4–16	39% 4–6 years; 73% 13–15 years
Thailand (27)	DENV-1–4 (1998–1999)	Fever	54 (331)	2.7%; 5.8%	7–11	ND
Thailand (30)	DENV-1–4 (1998–2002)	Fever	56 (569)	6.4%; 25.5%	7–15	ND
Thailand (45)	DENV serotype not known (2000–2001)	Fever	85 (34)	2; 7 (/100 person-years)	Birth – 8	ND
Thailand (32)	DENV-4 (2004–2005) and DENV-1 (2006–2007)	Fever	65 (535)	1.3–4.4%; 6.9%	4–15	ND
		Fever or other	20 (119)	11.8%; 16%^a^	0.5–15	
Thailand (29)	DENV-4 (2004–2005)	Fever	52 (27)	6.0%; 12.4%^a^	0.5–15	ND
Thailand (60)	DENV-1 (2001)	Clinical consultation	94 (54)	0.2%; 3.1%	All	6%
Thailand (28)	DENV-1 (2000–2001); DENV-2 (2002)	Clinical consultation	91 (733)	0.3%; 1–27%	All	ND
Vietnam (35)	DENV-2 (2004–2005); DENV-1 (2006–2007)	Fever ≥38° for ≥2 days	80 (953)	17–40/1000; 8–14%	2–15	20–29%
Vietnam (44)	DENV-1 dominant	Fever	90 (10)	0.2; 1.7 (/100 person-years)	Birth – 2	ND
Indonesia (37)	DENV-1–4 (2001–2003)	Fever	47 (17)	1.5%; 2.5%	All	ND
Indonesia (36)	DENV-1–4 (2000–2002)	Any	75 (74)	18; 74 (/1000 person-years)	18–66	ND
Philippines (46, 47)	DENV-3 and DENV-2 (2007–2009)	Clinical consultation	90 (115)	8–16; 103 (/1000 person-years)	Birth – 1	ND
Multi-centric (43)	DENV-1, -2, -3 (2006–2007) Southeast Asia	Fever	85 (20) Southeast Asia	0.9%; 6.1%^b^	>24 months	ND
	DENV-1, -3 (2006–2007) Latin America		63.2 (19) Latin America	8.3%; 22%^b^		

^a^Indicates positive index clusters only; cluster contacts followed for 15 days or

*^b^7 days*.

## Retrospective and Outbreak Studies

There were 12 retrospective or outbreak studies with measures of seropositivity and subjective recollection of fever and/or dengue-associated symptoms (Australia 1, British Virgin Islands 1, Colombia 1, Cuba 2, Puerto Rico 2, Brazil 2, South Pacific 1, Singapore 1, and Taiwan 1) (19–25, 53–57). In Cuba, the inapparent rate during the 1981 DENV-2 epidemic was estimated to be 71% in whites and 88% in blacks; the infections were considered likely to be 2°, as 45% of the population was thought to have seroconverted during the 1977 DENV-1 epidemic (54). During the 1997 Cuban DENV-2 epidemic, all 2° infections were clinically overt, but only 3–6% of 1° infections were apparent (55). In the Brazilian studies, inapparent rates were 27 and 53% in 1° infections vs. 37 and 39% in 2° infections (22, 23). In Colombia, repeated cross-sectional studies were carried out over a period of 17 months; 259 of 3,189 individuals showed clinical signs of viral infection and/or anti-DENV IgM antibodies; 86% were inapparent (24). In the Puerto Rican studies, where infections were majoritarily 1°, inapparent rates were 53 and 43% (56, 57). The Singapore and South Pacific studies were carried out during the epidemic period, and inapparent rates of 78 and 60% were reported, respectively (25, 53). The Australian study was performed in 1995 to address the 1993 DENV epidemic; only 11% of infections were considered to be inapparent (19). Longer-term recollection of 1° infections in individuals hospitalized in Taiwan with 2° infections suggested that 80% of 1° infections had been inapparent (21). Finally, returning US volunteers from the British Virgin Islands in a community with a suspected dengue case revealed that all DENV IgM-positive individuals had recollection of symptoms (20).

## Non-Residents (Expats, Military, and Travelers)

Nine prospective studies were identified involving expats, travelers, or military personnel staying in dengue-endemic areas (Haiti 1, Singapore 1, Somalia 1, Thailand 2, various 4) (48–51, 61–65). Seroconversion rates were low, yielding relatively few infections in any study. In the majority of studies, symptomatic infections referred to the occurrence of any dengue-like symptom. Sharp et al. (62) and Cobelens et al. (49) defined a symptomatic infection as fever plus any other symptom. Baaten et al. (50) obtained objective measure of fever or any other symptoms for defining symptomatic DENV infections (50). For the majority of individuals, the infection was considered to be their first, and the inapparent rate ranged from 0 to 100%. The majority (80–100%) of Americans (61) and Japanese (48) in Thailand had symptoms, whereas no Australian travelers to Asia reported symptoms (51). Sixty to 80% of Dutch travelers (world-wide) reported no symptoms (49, 50), whereas 50% of Israeli travelers had symptoms (63). Over 90% of Chinese workers experienced symptoms in Singapore (64), 85% of American military personnel had symptoms in Somalia (62), and all seven missionaries who were seropositive for DENV returning from Haiti reported dengue-like symptoms (65).

## Birth Cohorts – Maternal Antibody Studies

Four birth cohort studies addressed the potentially deleterious effects of maternal antibody for outcome of DENV infection in infants (Philippines 2, Vietnam 1, Thailand 1) (44–47). The occurrence of severe disease in infants following their first infection was noted by Halstead and colleagues and contributed to the development of the theory of antibody-dependent enhancement (ADE); antibodies from a first infection are insufficient to neutralize virus from a second infection of a different serotype and actually increase virus internalization in Fcγ receptor-bearing target cells and hence viremia (67). In infants, following a period of protection by maternally acquired anti-DENV antibodies, catabolism of these antibodies was hypothesized to decrease the titer of maternally acquired antibodies to enhancing levels and thus lead to the high incidence of severe dengue disease observed in infants (68). The birth cohort studies did not confirm the ADE hypothesis, although samples sizes were small. The inapparent DENV infection rate ranged from 75 to 90%.

## Prospective Studies

Twenty-three published papers describe analyses of prospective studies carried out in Southeast Asia (Thailand 10, Vietnam 1, Indonesia 2) (26–37, 60) and the Americas (Brazil 1, Nicaragua 5, Peru 2, Puerto Rico 1) (38–42, 52, 58, 59, 66) and one multi-center study covering Vietnam, Cambodia, French Guiana, and Brazil (43). The Thai studies occurred in one of three sites (Bangkok, Chang Mai, or Kamphaeng Phet); the Nicaragua, Peru, and Indonesia studies occurred in Managua, Iquitos, and Jakarta, respectively. The studies used one or more of several protocols: community-based cohort with paired healthy samples and laboratory work-up of suspected dengue cases/undifferentiated febrile illnesses, follow-up of school-based absenteeism, and index cluster analysis. Anti-DENV antibodies were detected using rapid diagnostic kits, in-house ELISA assays, hemagglutination inhibition assay, and/or neutralizing antibody tests. The age group sampled varied considerably, as did the seroprevalence and force of infection. Notably, although the majority of studies focused on children or the general community, one of the Indonesian studies targeted an adult cohort (36). The classification of a symptomatic DENV infection varied from the presence of any symptom, to just fever or fever plus two additional dengue-associated symptoms according to the WHO case definition. The inapparent DENV infection rate ranged from 20 to 97%; the weighted mean inapparent rates of cluster and cohort studies were 37 and 76% respectively. Mean inapparent rates in cohort studies were 77.1% in the Americas and 74.4% in Asia.

## Experimental Inoculations (Pre-1960s)

The experimental infection studies in the first half of the twentieth century provide the foundations of our current knowledge of dengue (69, 70). Siler et al. (69) conducted a series of induced infection experiments in military personnel using infected mosquitoes. Of 47 subjects, four individuals remained refractory to infection (or were asymptomatic) and two had very mild symptoms. The inclusion criteria aimed to recruit individuals who were naïve to dengue, but it could not be ruled out that some individuals had been previously exposed to DENV. Thus, assuming no immunity, the inapparent rate was at most 13% (6/47).

Simmons et al. (70) gave detailed accounts of the course and outcome of infection using mosquito-induced infections in American military personnel, residents of the Philippines, monkeys, and other animals (70). All 81 infections induced using competent species of mosquitoes (*A. aegypti*, *A. albopictus*) after an appropriate extrinsic incubation period (>9 days) yielded DF; only 13.6% were classified as mild (undefined). The extent of asymptomatic or even inapparent infections was clearly very low in American military personnel with no likely previous exposure to dengue. Adult Philippine individuals living in endemic zones for dengue proved immune, whereas those from non-endemic areas proved susceptible and were symptomatic following DENV infection. Successful experimental infections without symptoms in naïve Macaque monkeys were achieved, as demonstrated through onward transmission to mosquitoes. Although such onward transmission studies were not carried out in purportedly unsuccessful DENV-induced infections in humans, the authors state “In addition, it is quite probable that mild unrecognizable infections may occur in many adults, as has been proved possible in monkeys, and that virus can be transmitted from these apparently symptomless cases of dengue.”

## Potential Explanations for Differences in Observed Inapparent Rates

### Detection methodology

Retrospective surveys involving questionnaires of perceived symptoms are open to perception bias as well as the non-specificity of dengue symptoms. Prospective studies use varying definitions of a symptomatic dengue episode and different protocols for case detection that generate considerable variation in inapparent infection rates. This is particularly well demonstrated by two alternative protocols implemented in the same population: the index cluster approach revealed that many inapparent infections, as defined by school absenteeism and passive case detection, had fever or other symptoms (32). In addition to the increased case detection sensitivity of the index cluster approach, such an approach may also suffer from ascertainment bias: viruses responsible for index cases identified by clinical presentation may be more pathogenic and thus lead to increased symptomatic infections in the clusters than would occur in the general population. However, symptomatic cases in clusters were found to be milder than those in the cohort study (33).

### Influence of human genetics

A broad overview of global incidence of disease attributable to dengue suggests that disease severity is greater in Southeast Asia than in the Americas and that severe dengue is infrequent in Africa (71). One major confounding factor is separating geography and the environment from ethnicity. However, the dengue epidemics in Cuba have given support to the hypothesis that individuals of African ethnicity are less susceptible to disease than white Caucasians (54). There is increasing evidence from candidate gene and genome-wide studies that human genetics play a role in the outcome of infection (72–74). Only one study, however, has attempted to assess the impact of human genetics on inapparent outcome of infection. A polymorphism in the FcγRIIA was found to be associated with inapparent infection vs. DF or DHF in the Cuban population (75). In light of the epidemiological observations on the global variation in the incidence of DENV infection and severe disease, it seems likely that at least some of the observed variation in the inapparent rate is attributable to human genetics.

### Primary vs. secondary vs. post-secondary infections

**Figure 1:**
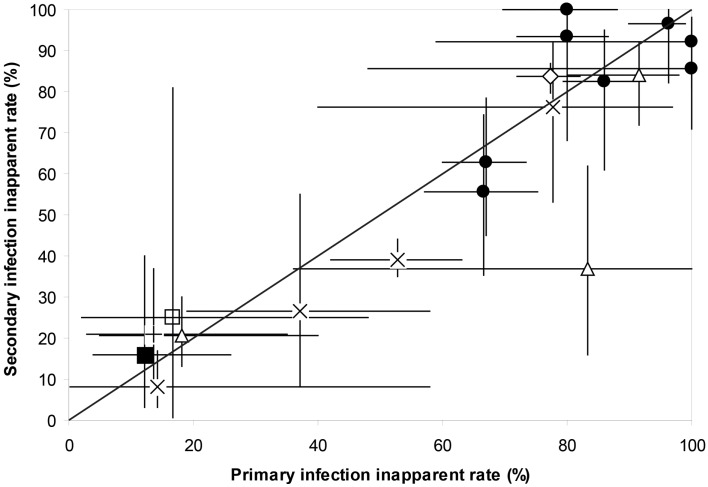
**The inapparent rate of primary and secondary DENV infections within the same study site for Nicaragua (39, 41, 42, 59), Thailand (26, 29, 32), Brazil (22, 23, 38), Peru (52), French Guiana, Cambodia, and Vietnam (43)**. Shown are means and 95% binomial confidence intervals. Superimposed is the line of equality where the inapparent rate of 2° infections equals that of 1° infections. ● Nicaragua, ■ Vietnam, + Cambodia, × Brazil, ♢ Peru, □ French Guiana, ∆ Thailand.

Secondary infections are considered to result in more severe outcome of infection, due to the phenomenon of ADE and/or cross-reactive T cells (67). Very little, however, is known specifically about the impact of previous exposure to two serotypes on the outcome of infection with a third serotype. A cohort study in Brazil found that there were significantly more inapparent infections in 1° as compared to 2° infections (22). Olkowski et al. (66) found that in Peru there was a reduction in symptomatic outcome in post-secondary infections compared with 1° and 2° infections as defined by pre-infection serological profile; 93% reduction in symptomatic outcome for DENV-3 and a 64% reduction in disease outcome for DENV-4 (66). However, Montoya et al. (42) found no differences in inapparent infection rates in first, second, third, or even post-secondary infections in Nicaragua (42). Several studies measuring the inapparent rate also evaluated whether the observed infection was 1° or 2°(22, 23, 26, 29, 32, 38, 39, 42, 43, 52, 59). A mixed model logistic univariate regression revealed that although there were significant differences in inapparent rate among studies, there was no significant difference within study site between the inapparent rate in 1° and 2° infections (Wald’s $\chi_{1}^{2}=0.27$, *P* = 0.61) (Figure 1). Too few studies have been able to address post-secondary (third or fourth) infections and infection outcome for any meta-analysis to be performed.

### Short-term cross-protective immunity

Sabin set the foundations for our current appreciation of acquired immunity to dengue and the extent of cross-immunity (76). Three important results arising from these early studies are of pertinence here: (i) there exists a minimum infective dose, which could lead to no symptoms but partial immunity; (ii) immunity to a recent previous infection alters the outcome of a subsequent infection; and (iii) virus attenuated via mouse passage yields symptomless infections that are transmissible to mosquitoes, albeit poorly so. For cross-immunity, active immune protection was achieved for up to 2 months, slight malaise/fever occurred in 2° infections 2–3 months later, and even after 9 months, dengue episodes were milder. DENV infection and onward transmission to mosquitoes was demonstrated in 2° infections at 2–3 months and 9 months post-1° infection. Recent statistical and theoretical modeling approaches lend support to Sabin’s demonstration that there exists cross-serotype non-sterilizing immunity resulting in milder clinical symptoms that may last for up to 2 years (77–79).

**Figure 2:**
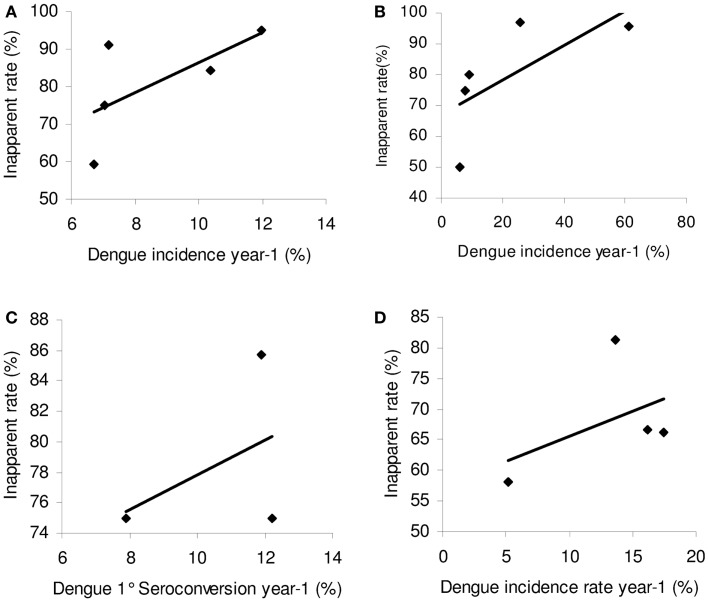
**The relationship between the inapparent infection rate and DENV infection intensity the previous year**. Linear trend lines were fitted. **(A)** Nicaragua (39, 41) *R*^2^ = 0.43; **(B)** Peru (52) *R*^2^ = 0.477. **(C)** Vietnam (35) *R*^2^ = 0.20; **(D)** Thailand (30) *R*^2^ = 0.21.

Endy et al. (30) first noted a significant impact of the previous year’s dengue incidence on the inapparent rate; a high incidence the previous year increased the current year’s inapparent rate. This was proposed to be a result of heterotypic cross-immunity, as described above. A plot of the inapparent rate against previous dengue incidence reported in the longitudinal cohorts with sufficient data (Nicaragua, Peru, Thailand, and Vietnam) (30, 35, 39, 41, 42, 52), all show the same positive relationship between the incidence of infection the previous year and the inapparent rate in the current year (Figure 2). However, the strength of the relationship seems proportional to the seroprevalence. In Nicaragua and Peru, the seroprevalence in the population was high, with most children having been exposed to at least one serotype by 10 years of age. In Vietnam, by contrast, the seroprevalence was lower for the same age group (35). The correlation of the incidence of infection the previous year and the inapparent rate in the current year are strongest for areas of higher seroprevalence and thus where the majority of new infections are 2°. It is notable that the positive relationship for inapparent rate and previous year DENV incidence of infection was significantly stronger when considering incidence of disease the previous year (*R*^2^ = 0.76) rather than infection (*R*^2^ = 0.2) in the Vietnam cohort. This raises the question of whether the acquired immune response is stronger when infection is accompanied by disease, rather than being asymptomatic; this has previously been observed in Japanese Encephalitis (quoted in Barnes and Rosen) (53).

**Figure 3:**
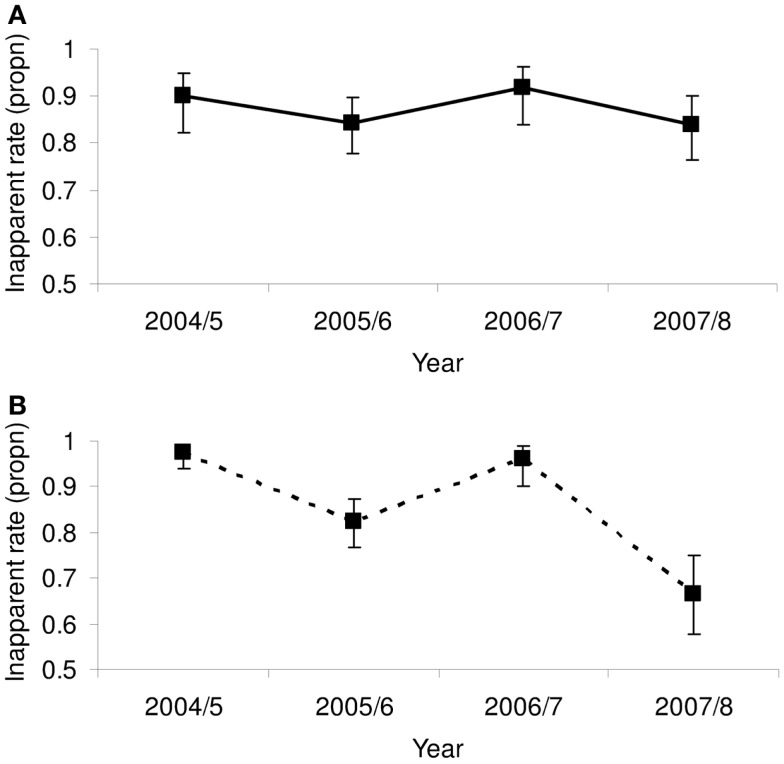
**The proportion of (A) 1° and (B) 2° infections in the Nicaraguan Pediatric Dengue Cohort Study (39) that was inapparent**. Shown are the proportions and the 95% binomial confidence intervals.

Of key importance is to ascertain whether the inapparent rate is indeed driven by heterotypic immunity. The Nicaraguan Pediatric Dengue Cohort Study throws some light on the question, differentiating 1° from 2° infections (39). A reduced symptomatology in the outcome of infection through heterotypic immunity would only apply to 2° infections. The fluctuations in the inter-annual inapparent rate in 2° infections oscillated inversely with the previous years’ incidence rate, ranging from 67 to 97% and significantly so with non-overlapping 95% binomial confidence intervals (CIs) (calculated as inapparent/total infections) (Figure 3B). By contrast, although the same oscillating inter-annual pattern was observable in 1° infections, for which cross-protective immunity is not relevant, the fluctuations were dampened, ranging from 82 to 92%, with overlapping 95% CIs (Figure 3A). This gives credence to the hypothesis that short-term cross-protective immunity plays a significant role in determining infection outcome in 2° infections.

More recently, several studies have analyzed the importance of the time interval between successive DENV infections on the outcome of infection in first, second, and post-2° infections. In the Nicaragua cohort, Montoya et al. (42) found that the time interval between successive first and second infections leading to an inapparent second infection outcome was significantly shorter (1.8 years) than that leading to a symptomatic infection outcome (2.6 years) (42). There was no impact of time interval for post-2° infections. In Thai cohorts, Anderson et al. (34) found similar results; there was a higher probability of an infection being inapparent in 2° infections if occurring within 1.4 years of a previous (thus 1°) infection; the time interval between infections leading to DF or DHF was longer, at 1.9 and 2.6 years respectively, although the number of DHF was small (34). Again there was no difference for post-2° infections.

### Impact of the current year’s incidence

Endy et al. (30) also noted a significant negative impact of the current year’s dengue incidence on the inapparent rate; a high concurrent incidence reduced the inapparent infection rate (30). This relationship was confirmed in the same cohort (*R*^2^ = 1) (32), but to a much lesser extent in both the Nicaraguan and Vietnamese studies (*R*^2^ = 0.12 and 0.23, respectively) (35, 39). There was no relationship in the Peruvian study (*R*^2^ = 0.07) (52). Careful studies taking into account the force of infection in relation to inapparent and symptomatic infection are needed.

The interplay between short-term cross-protective immunity and spread of a novel serotype will lead to inapparent rates that will depend on the historical prevalence of dengue (thus, the extent of 2° infections), the recent incidence of dengue (cross-protection), and the nature of the virus itself. By example, in the Nicaraguan cohort, the expansion of DENV-2 in 2005 (increasing from 20% of infections to 53%) was accompanied by an increase in transmission intensity (8.6–11.1% incidence) and increase in symptomatic outcome, particularly for 2° infections (3–25%) (39). This suggests a classic reaction to a new serotype in a background primed by other serotypes. The following year, transmission intensity dropped (from 11.1 to 5.8%), there was an increase in the predominance of DENV-2 (90% of all infections), and a decrease in rate of symptomatic infections; this decrease was most dramatic for 2° infections (25 to 4%), suggesting an important influence of cross-protective immunity. Then, transmission intensity increased with a concomitant rise in disease severity in both 1° and 2° infections. The rise in disease severity despite no change in serotype would suggest that the virus had evolved. Indeed, there was a clade change in 2006 (from DENV-2 clade 1 to clade 2B), which was associated with increased severity in the cohort and which could have contributed to the final increase in severity of infection outcome (80).

Morrison et al. (52) proposed a three-step chronology for the invasion of a novel serotype: amplification, replacement, and epidemic (52). Implicitly underlying this chronology is the notion of viral adaptation to its novel environment in competitive circumstances. Viral evolution may also contribute to the rise in the inapparent rate following the epidemic phase. Abortive dengue epidemics, where the incidence of disease is low, have been noted previously, and islands in the South Pacific have escaped severe outbreaks occurring in their neighbors (53, 81).

### Viral genetics

The importance of viral genetics in determining the outcome of infection has been suggested in the context of 1° vs. 2° infections (82), severe primary epidemics in naïve populations (53, 81), molecular variants yielding high replication rates in the laboratory (83), DENV serotype, genotype, and clade, sequential order of serotypes in infections (84), and interaction with pre-existing serotype-specific immunity (80, 85). However, there is no consistent pattern. Whilst confounding factors influence the disease severity of an epidemic, the genetic diversity of the viruses likely plays an important role. As with all arboviruses, the RNA-dependent RNA polymerase’s lack of proofreading activity coupled with the large virus population sizes lead to the constant generation of variants. This means that RNA viruses generally circulate as dynamic mutant networks (86). Recent studies reveal that DENV exist as heterogeneous populations in patients and mosquito vectors (87–93), but the significance of this is unclear. Coupled to the selective forces in both host and vector, these features would enable the circulating viral population to change significantly even during the course of a single epidemic. Indeed, the proportion of severe cases has been reported to increase toward the end of an outbreak (94–96). Endy et al. (27) also noted that symptomatic cases extended later into the season than inapparent infections (27). DENV populations may also rapidly change because of periodic selective sweeps and by intra-serotypic recombination (97, 98), though this latter remains contentious. DENV infection leads to a spectrum of outcome severity from inapparent to mild or severe disease; small changes in viral genetics could lead to significant changes in infection outcome. However, despite increasing evidence for a role of viral genetics in the outcome of infection, to date we do not know the extent to which the observed variation in the inapparent rate is influenced by viral diversity and evolution.

Genetic variation has been associated with differences in virus transmission efficiency (99, 100). Viral adaptation during its invasion phase may improve transmission capacity to mosquitoes responsible for an epidemic and/or result in strains that are responsible for more severe cases toward the end of the epidemic. Infectiousness to mosquito increases with viremia and although hospitalized cases have higher viremia, symptomatic but ambulatory cases infected mosquitoes equally well (101). Currently, we have no knowledge about the comparative transmissibility of inapparent infections, a crucial element that needs to be addressed.

## Conclusion and Implications

Establishing risk factors and the extent to which DENV infections are inapparent is important not only for assessing whether there will be a silent invasion of DENV into hitherto unaffected areas but also for improving our understanding of dengue epidemiology and infection severity. The epidemiological evidence to date suggests that whilst the majority of infections are inapparent in endemic settings, there are recognizable patterns that are consistent with an important role for short-term heterotypic non-sterilizing immunity. However, similar inter-annual fluctuations in the inapparent rate in 1° infections require additional explanations, potentially suggesting a role for viral evolution. One intriguing avenue of research is the extent to which heterotypic immunity promotes viral diversification. Likewise, it would be interesting to assess how human genetics impacts upon infection outcome.

Extrapolating from endemic settings to an invasion scenario may not be applicable, especially given the significant role seemingly played by the immune response in 2° infections, whether enhancing or protective. Retrospective serological surveys in recent virgin soil epidemics, for example, in Madeira and Cape Verde, would provide invaluable information on the extent of inapparent infections under such scenarios and give a better idea of what to expect under invasion and hence how best to implement surveillance and control efforts.

Finally, an appreciation of inapparent DENV infections is important for both interpretation of vaccine trials and vaccine uptake. It has been suggested that, in the light of the high incidence of inapparent DENV infections, vaccine trials should consider other measures of vaccine efficacy in addition to occurrence of clinically apparent infections, i.e., to consider efficacy against infection and not just disease (102). Moreover, pre-existing immunity from prior symptomatic and inapparent DENV infections will likely affect the type of immunity induced by tetravalent vaccines (i.e., homotypic vs. heterotypic), and this needs to be considered as well. Lastly, successful vaccination should reduce the large reservoir of inapparent infections that are likely capable of onwardly transmitting the virus, thus further reducing DENV transmission.

## Author Contributions

All authors contributed equally to the manuscript. Specifically, Richard Paul and Eva Harris conceived the study. All authors participated in the data acquisition and preliminary writing. Etienne Simon-Loriere created the graphics and Richard Paul carried out the statistical analyses. Richard Paul and Eva Harris wrote the final version and all authors read, corrected, and approved the final version.

## Conflict of Interest Statement

The authors declare that the research was conducted in the absence of any commercial or financial relationships that could be construed as a potential conflict of interest.

## Supplementary Material

The Supplementary Material for this article can be found online at http://www.frontiersin.org/Journal/10.3389/fimmu.2014.00280/abstract
