# Supplementary material for: Epidemiological Risk Factors Associated with High Global Frequency of Inapparent Dengue Virus Infections
Source: Front Immunol. 2014 Jun 11;5:280. doi: 10.3389/fimmu.2014.00280 (PMC4052743; doi:10.3389/fimmu.2014.00280)
Supplement: Supplementary file 1 [file Data_Sheet_1.DOC]

**Supplementary information Grange et al.**

**More detailed description of studies documenting inapparent DENV infection rates.**

**Epidemiological studies**

**Retrospective and outbreak studies**

***South Pacific***

1. Barnes and Rosen (1974) performed a retrospective serological and questionnaire survey on dengue symptoms in Niue Island, Polynesia to investigate the DENV2 1972 epidemic (1). There had been no dengue epidemic for 25 years and this was supported by the much smaller number of high (>80) hemagglutination-inhibition (HAI) titers in individuals under 25 (3%) than in those over 30 years of age (>80%). In those less than 25 years old (N=375), there were 110 individuals who reported having symptoms. In contrast, serology carried out in 242 individuals less than 25 years old revealed that 89% were DENV1 or DENV2 seropositive. This yields an estimated 60% inapparent infections.

***Australia***

2. A retrospective serological and questionnaire survey was performed on 1000 individuals in Queensland, Australia to assess the impact of the 1993 DENV2 epidemic (2). In 139 individuals, aged less than 50 years old and seropositive for DENV2, 123 had recollection of symptoms (88.5%). No symptoms were specific to dengue seropositive individuals, and although fever was very common (95% of seropositive cases) taste change, rash and joint and bone pain were more differential between seropositive and negative groups. Only 23 individuals in the 1000 surveyed had recollection of having had symptoms during the DENV1 epidemic 12 years earlier; nine of these had DENV2 infection and five with hemorrhage. The impact of the 1981 epidemic on the population is considered small and thus most infections in the 1993 might be considered as 1° (primary) infections.

***Cuba***

3. Cuba has had sporadic epidemics of dengue over the last 30 years. In 1977, a DENV1 epidemic swept through the population and led to an estimated seroconversion of 45% of the population during a period of 24-36 months (3). In 1981 a DENV2 epidemic resulted in 344,203 dengue cases, 10,312 DHF (WHO Grades 3 & 4) cases and 158 deaths in a period of 4 months (4). A retrospective serological survey performed in Havana coupled with a questionnaire and estimated that the clinical:subclinical ratio was 1:2.5 in whites and 1:7.4 in blacks (3). Twenty-three percent of the population had seroconverted to DENV2.

4. From 1982-1996 a program of intense vector control was implemented. In 1997, a DENV2 epidemic occurred in Santiago de Cuba. A retrospective serological study in 1,151 individuals across all ages found 59.1% seronegative, 24.8% seropositive for DENV1, 6.3% seropositive for DENV2 and 9.9% seropositive for both (5). In children under 16 years of age, who could not have experienced the 1981 DENV2 epidemic, only five of 251 (2%) were seropositive for DENV2. An estimated 4,800 adults had a 2° (secondary) infection on top of a DENV1 infection (acquired 20 years previously). All 2° infections were clinically overt. Only 3-6% of 1° infections were estimated to be clinically overt based on the incidence of diagnosed cases and cohort seropositivity.

***Puerto Rico***

5. Likosky et al. (1973) studied the impact of the 1969 DENV2 epidemic in Puerto Rico. Combining information about clinical presentations with serological surveys, 43% of infections were inapparent (6). Acute illness infections were categorized as confirmed cases (4-fold increase in HAI titers between 1^st^ and 2^nd^ samples, or in children <6 years old, the presence of anti-DENV antibodies even if only one serum sample was available), presumptive (HAI titer ≥1:640 without a 4-fold change in titer between paired samples) and negative (without antibodies, e.g. HAI titer <1:10). Apparent infections were those classified as serologically positive with dengue-related symptoms.

6. Rodriguez-Figueroa et al. (1995) performed a serological and questionnaire study shortly after the dengue season of 1991 (7). DENV2 was the circulating virus. Any dengue-related symptoms were considered. Using MAC-ELISA, 53% (31/59) of infections were inapparent. 49 of the 59 infections were 1° infections.

***Virgin Islands***

7. Lyerla et al. (2000) conducted a retrospective study of incidence of dengue in American volunteers in a community-assistance program in the British Virgin Islands who developed symptoms consistent with dengue (8). A questionnaire was used to assess whether the individuals could be classified as having clinically diagnosed dengue (fever plus 2 of the classic dengue symptoms). Twenty-two (of 32) individuals met the clinical case definition. Fifteen of these provided a serum sample and all were IgM-positive. Of the ten individuals who did not meet the clinical case definition, six provided a serum sample and all were IgM-negative. There were no inapparent infections identified.

***Brazil***

8. Vasconcelos et al. (1998) performed a cross-sectional seroprevalence and symptom (any dengue-related) study during a DENV2 epidemic in Fortaleza across all age groups (9). Positive HAI titers < 1:1,280 were considered as 1° infections, while titers ≥ 1:1,280 were considered 2° infections. 1,341 samples were tested of which 589 (44%) were dengue-positive. Of these, 243 reported no symptoms (41%). Of the 93 1° infections, 53% were inapparent, whereas only 39% were so in the 2° infections.

9. Rodrigues et al (2002) performed a cross-sectional serological study (MAC-ELISA) in inmates (N=105; age 14-18 years) and staff (N=91; age >20 years) in 1997 in the State of Sao Paulo during the epidemic season with a questionnaire on the occurrence of classical dengue symptoms (10). Forty-two individuals were IgM-positive, of which 15 were also IgG-positive. Of the twenty-seven individuals with presumably 1° infections, ten did not complain of any symptoms, and of the 15 presumably 2° infections, four did not complain of any symptoms, yielding an inapparent rate of 27% and 37% in 1° and 2° infections respectively.

***Colombia***

10. Mendez et al. (2006) conducted a prospective study on a random sample of 100-150 individuals (age 7-20 years) every three months in four cities from November 2002 to March 2004 (six visits) (11). The first three visits occurred during an on-going epidemic (all four serotypes recorded but with predominance of DENV2 and DENV3 in last three visits). The study detected virus by isolation using the C636 *Aedes* *albopictus* cell line followed by immunofluorescence (IFA) using specific monoclonal antibodies. Serological evidence of a recent infection was assessed by IgM ELISA. A questionnaire on recent occurrence of dengue-related symptom was implemented. Viral isolation was confirmed in 6.7% (215) of 3,189 participating individuals. Only 14% of these reported symptoms (mostly fever, rash and hemorrhage).

***Taiwan***

11. A hospital based study (2006) of 135 dengue patients found that 55 were secondary infections, 43 (78%) of whom recalled having had no symptoms during the first infection, likely from the 1988-1991 DENV1 epidemic (12).

***Singapore***

12. Yap et al. (2013) carried out a cross-sectional seroepidemiological study in dengue outbreak areas during the 2007 epidemic. Blood samples were taken from 3,939 individuals of all ages and tested with the Panbio IgM and IgG ELISA (13). Individuals were interviewed for subjective recollection of fever and/or dengue associated symptoms. There was evidence of a recent infection in 6.8% of individuals, but 78% of these did not recall symptoms. Of the 59 individuals with recollection of symptoms, only five had consulted a clinician and only one was diagnosed with dengue. Individuals older than 45 years of age were more likely to have an inapparent infection than younger individuals (88% *vs*. 63%).

**Prospective studies**

***Puerto Rico***

13. Waterman et al. (1985) performed a paired (before/after “dengue season”) serological survey coupled with a questionnaire on occurrence of fever during the 1982 DENV4 epidemic (14). Using case definitions based on fever and a four-fold rise in HAI titre, the percentage of inapparent infections was estimated to be 45%.

***Nicaragua***

14. Balmaseda et al (2006) reported a two-year study in 2001-2003 based on school absenteeism in approximately 1,000 children aged 4-16 using two days of absenteeism as a marker for dengue risk (15). The dominant serotypes were DENV2 and DENV1 in the first and second years respectively; DENV1, DENV2 and DENV4 (to a lesser extent) were co-circulating and DENV3 circulated prior to this time. Seroprevalence rates against DENV were 75% at the age of four and reached 100% at age 16 in this cohort in Managua. Fever plus any classical dengue-related symptoms were used to identify suspected dengue cases. In 2001, there were 39 seronegative individuals of whom seven seroconverted and none showed symptoms. There were 52 secondary cases of which four showed symptoms. In 2002, there were 70 seronegative individuals of whom five seroconverted and none showed symptoms. There were 42 secondary infections of which six were symptomatic. Overall, inapparent infection rates of 93% and 87% were observed.

15. Balmaseda et al. (2010) carried out a prospective pediatric cohort study of dengue (currently on-going), of which the first four years are described (2004-8). 3,800 children from 2-9 years of age were recruited in 2004 and then every year new two-year olds were recruited (16). The study design involved passive surveillance of febrile presentations that were tested for acute DENV infection if meeting with WHO criteria for dengue syndromes or undifferentiated fever. Acute samples were tested by RT-PCR and virus isolation, and acute and convalescent paired samples were tested for IgM antibodies (MAC-ELISA) and for total anti-DENV antibodies (Inhibition ELISA). Every July, prior to the dengue season, blood samples were collected from participating individuals to test for dengue serological status (inhibition ELISA for total anti-DENV antibodies). Children with paired annual samples showing a ≥4-fold increase in DENV-specific antibody titer but without clinical presentation for dengue in that year were considered inapparent DENV infections. Over the four-year period the force of infection (as deduced by the incidence of DENV infections and the age-specific seroprevalence rates) decreased. There was a gradual replacement of DENV1 by DENV2. The inapparent rate oscillated between 2004/5 and 2007/8 (18:1, 5:1, 16:1, 3:1). The inapparent rate was lower in primary infections (compared to secondary infections) in years of high inapparent rates (2004/5 and 2006/7) and lower in secondary infections in alternate years (2005/6, 2007/8).

16. Reyes et al. (2010) performed an index case household cluster study in the same study site as the pediatric cohort described above (17). In 2006 and 2007, when the pediatric cohort identified inapparent infection rates of 94% and 75% respectively, the cluster study identified five asymptomatics of the 12 DENV positive cases. In the first year all four DENV infections were asymptomatic, whereas in 2007 only one of eight was asymptomatic. The contrasting patterns in the cluster and cohort study may not be directly comparable, notably because of the different ages of the participating individuals. In addition, the criteria for defining a suspected dengue case required fever plus two of the classical WHO criteria. Asymptomatic infections were infections with no symptoms. There were cases of undifferentiated fever that were DENV-positive.

17. Gordon et al. (2014) provide an update of the Pediatric Dengue cohort study described in (16) extending to 5,545 children aged 2-14 years old (18). The incidence of dengue infections oscillated from 67 to 110.1 infections per 1,000 person-years and the incidence of cases from 3.4 to 43.5 per 1,000 person-years. The inapparent rate oscillated from 60-95%. DENV2 replaced DENV1 over the period 2004-7 and then DENV3 became dominant from 2008. There were few mixed DENV infections.

18. Montoya et al. (2013) performed a risk analysis for inapparent infection outcome in the Nicaragua cohort, extended from 2004 until 2011 (19). The criteria for sero-conversion, definition of infection outcome are as above (16). In addition, serotype-specific neutralizing antibody levels were measured using a Reporter Viral Particle-based assay in longitudinal samples from a subset of participants. Paired annual samples thus permitted classification of DENV infections as 1°, 2° or post-secondary. There were 341 paired infections with documented infection outcome and knowledge of infection number (1°, 2° or post-secondary). Of 162 2° infections, 117 were inapparent in both 1° and 2° infections; the mean time interval between these infections was estimated at 1.8 years. Thirty-two paired DENV infections were inapparent 1° and symptomatic 2°, occurring at a mean time interval of 2.6 years. Thirteen paired DENV infections were successively symptomatic (1°) and inapparent (2°), occurring at a time interval of 2.2 years. By contrast, of 169 post-secondary infections, the comparative time intervals were 2.7 years (inapparent 1° & 2°, N=103), 2.7 years (inapparent 1°, symptomatic 2°, N=53) and 2.2 years (symptomatic 1°, inapparent 2°, N=13).

***Brazil***

19. Honorio et al. (2009) performed a community-based study of seroconversion rates in households from three areas of Rio de Janeiro in 2008 when there was a severe dengue epidemic (majoritarily DENV2) that led to 330,000 cases and 240 deaths (20). Paired samples collected from 337 individuals of all ages from 171 households before and during the epidemic period were tested using the Panbio IgG/IgM RDT and RT-PCR. Primary infections were defined as those which were IgG-negative in the first sample and IgM-positive in the second sample; secondary infections were IgG-positive in the first sample. The seroprevalence rate, established using the pre-epidemic samples, varied from 56 to 77% in the three study areas. Symptomatic infections were defined as those with fever (>38°C) plus at least two WHO symptoms. There were 30 recent (IgM-positive) infections, of which 77% were inapparent and nine were primary infections.

***Peru***

20. Morrison et al. (2010) conducted a six-year passive surveillance community-based cohort study (1999-2005) in Iquitos where patterns of seroconversion were monitored every six months (21). This was combined with an active surveillance program based on school-absenteeism, with approximately 1,000 individuals recruited each year. To maintain an active cohort of 2,400 individuals, a total of 4,586 individuals were recruited over the whole time period. The majority (78%) were less than 18 years old. Baseline seroprevalence rates were high, with less than 20% having no serological evidence of exposure to DENV. Seropositivity increased from 60% in children aged five to 90% by the age of 14. Of 3,903 individuals that provided at least two samples, there were 1,414 seroconversions. The majority were 2° infections.

There had been previous DENV epidemics in Iquitos: DENV1 in 1990/1 and DENV2 in 1995. During the first 15 months of this study (thus 1999-2000), DENV1 & DENV2 co-circulated at low levels. In 2001, DENV3 was introduced and caused an epidemic. Seroconversion rates rapidly increased from five per 100-person-years in 2000/1 to 90 per 100-person-years at the height of the epidemic at the end of 2002, by which time DENV3 had replaced DENV1 & DENV2. The inapparent rate, however, during this time increased from 50% to 83%. After the peak of the epidemic, the inapparent rate increased steadily to 98%.

21. Olkowski et al. (2013) provide a continued description of the above cohort from 2006-2011(22). Of note, DENV3 dominated in 2006-8 and then DENV4 expanded and dominated from 2008. The same protocol was applied with some amendment. Notably symptomatic dengue was defined as fever plus one dengue associated symptom, or in some cases dengue symptoms without fever, with viral detection by IFA or RT-PCR or a four-fold rise in IgM in acute and convalescent sera. At baseline recruitment, individuals were classified into DENV-naïve, monotypic or multitypic using neutralization assay for each serotype: 70% relative plaque reduction at cut-offs of 1:60 for DENV1 & DENV3, 1:40 for DENV4 and 1:80 for DENV2. The study specifically addressed the extent to which there was a reduction in disease following post-secondary infections compared with 1° and 2° infections. There was a 93% reduction in disease outcome for DENV3 and a 64% reduction in disease outcome for DENV4, to which the population was considered largely naïve. For DENV3, there were 28 symptomatic cases of 109 1° infections, 16 of 107 2° infections and seven of 745 post-secondary infections. For DENV4 there were 35 symptomatic cases of 168 1° infections, 54 of 320 2° infections and 72 of 1107 post-secondary infections.

***Thailand***

22. Burke et al. (1988) performed a prospective paired sample serological survey on children 4-16 years old in Bangkok with suspected dengue identified through school absenteeism (two days absent)(23). Seroconversion was established by HAI and then PRNT. Symptomatic cases were defined by occurrence of febrile illness. Of 103 individuals who seroconverted during the 1980 dengue season, 90 were inapparent with less than one day of school absence. Infections in 43 of 47 DENV-naïve individuals were inapparent and a similar proportion (47 of 56) of individuals with pre-existing anti-dengue antibodies was also inapparent.

23. Endy et al. (2002) performed a prospective cohort study of primary school children (7-11 years of age) in northern Thailand with active case surveillance to note febrile illness, through school absenteeism (same day) and school nurse visits (24). Samples for serology (HAI and IgG/IgM ELISA) were taken in January, June, August and November. The cohort of approximately 2,000 children was recruited from 12 schools. Over a three year period, there were 168, 126 and 37 seroconversions of which 55%, 48% and 65% were inapparent.

24. In the same cohort and using the same surveillance approach, Anderson et al. (2011) reported on the effect of Japanese Encephalitis Virus (JEV) antibodies on the outcome of infection with DENV (25). Over a five-year period (1998-2002) there were 569 DENV infections, of which 55.5% were inapparent. The percentage of symptomatic cases varied by year, from a low of 26% to a high of 51%, in 2000 and 2001 respectively. The presence of JEV antibodies increased the risk of symptomatic outcome of infection (OR = 1.55), particularly in individuals having a 1° DENV infection (OR = 2.75).

25. Endy et al. (2011) described in detail the year-specific and school-specific variation in the inapparent rate noted above (26). The factors contributing significantly to the observed variation were: circulation of many serotypes and a high incidence of infection both decreased the inapparent rate; a high incidence the previous year increased the inapparent rate. Circulation of DENV3 was associated with a lower likelihood of a child having an inapparent infection.

26. Mammen et al. (2008) performed an index case cluster study in the same site as above (27). Overall, 556 children were enrolled as neighbors in 12 index DENV case positive and 22 DENV negative clusters. DENV infections in neighbors were ascertained by RT-PCR and IgG/IgM ELISA. Questionnaires on current health state and history of illness during past five days were used and oral temperature taken. Blood samples were collected on Days 0 and 15. There were 27 DENV infections of which 14 (50%) were inapparent (lack of objective or subjective fever). Inapparent infections were more likely to be 1° than 2° infections.

27. Yoon et al. (2012) describe a combined analysis of a four-year (2004-7) prospective cohort study that additionally served to identify index cases for household cluster analyses (28). This cohort is an extension of that described above. DENV4 was the dominant serotype in 2004 and 2005; DENV1 was the dominant serotype in 2006 and 2007. The prospective cohort revealed an inapparent rate of 71%, 74%, 53% and 69%. By contrast, the cluster studies revealed an opposite image; there was a majority of symptomatic infections clustered around the index case. Inapparent rates calculated from the cluster analyses differed for 1° and 2° infections, although the numbers were too low for 1° infections to be able to make any reasonably confident conclusions. Overall, the combined 1° and 2° infections yielded inapparent rates of 17%, 14%, 22% and 21%.

28. Yoon et al. (2013) contrasted the symptomatic cases identified in the above cohort and cluster studies (29). Symptoms were milder in the cluster dengue cases than the cohort dengue cases. Of 126 cluster DENV infections, 19% were asymptomatic. Of the symptomatic cluster dengue cases, only 66% had fever.

29. Van Benthem et al. (2005) performed a paired sample serological survey of 1,750 individuals of all ages in a community in the vicinity of Chiang Mai in 2001 (30). Recent infections were identified by IgM ELISA. Symptomatic infections were defined as those individuals consulting the medical practitioner and who were IgM-positive. Only three individuals of 113 who were seropositive consulted, yielding a 97% inapparent rate.

30. In the same study site, Vanwambeke et al. (2006), reported a similar inapparent rate with the same diagnostic criteria over five surveys carried out from 2001 until 2003; the inapparent rate varied from 65% (2003) to 99.7% (2002) (31). There were 596 seroconversions in two surveys of 2002 and 82 in the two surveys of 2003.

31. Anderson et al. (2014) analyzed the importance of time interval between 1° and 2° (and that between 2° and post-secondary) infections in schoolchildren recruited in the cohort studies of Endy et al. (2002) and Mammen et al. (2008) (covering years 1998-2002 and 2004-7 respectively) (32). Samples were collected quarterly. Subclinical infections were classified as those for which there was a four-fold rise in HAI titer against any of the four DENV serotypes and for which there was no record of a confirmed symptomatic DENV infection during the active surveillance period of that year (June to November or December for 1998-2002 and 2004-7 respectively). Children were classified as HAI-negative if titers <10 for all four serotypes; HAI-monotypic if HAI titer ≥10 for one serotype and HAI-multitypic if HAI titer ≥10 for two or more serotypes. Of 123 infections, 89 were subclinical, 27 DF and seven DHF. Of 39 1° infections (HAI-negative at prior sampling), 22 were subclinical, 12 DF and five DHF. Of 11 2° infections (HAI-monotypic at prior sampling), nine were subclinical and two DF. Of 73 post-secondary (HAI-multitypic) infections, 58 were subclinical, 13 DF and two DHF.

***Viet Nam***

32. Tien et al. (2010) followed an open school-based cohort (2-15 year olds) over four years (33). Through IgG seroconversion, the 1° infection rate was 11.4%. Using active/passive febrile case detection, dengue was suspected in case of fever ≥ 38°C for ≥ 2 days. Dengue infection was confirmed by RT-PCR, or viral isolation or NS1+ or IgM-positivity or a four-fold increase in IgG titer. 1° vs. 2° infections were defined according to the IgM:IgG ratio (1° ratio ≥1.8; 2° <1.8). The symptomatic to asymptomatic ratio was found to vary from 1:3 to 1:6 according to the year of study.

***Indonesia***

33. Porter et al. (2005) carried out a cohort study recruiting adult factory workers who were followed for illness (detected through absenteeism) and for inapparent infections over a period of two years through three-monthly serological analyses (using a random sample of 250 individuals who had not had symptomatic dengue)(34). Of more than 2000 individuals recruited (age 18-66 years), there were over 4,000 person-years of follow-up, yielding an estimated 18 cases per 1,000 person-years. Of the 250 random samples, 125 were from dengue-free districts and 125 from districts with dengue cases. There were 16 seroconversions with a four-fold increase in HAI titer, 14 of which were confirmed by PRNT. In the dengue-free district, there were 3 seroconversions with a four-fold increase in HAI titer, two of which were confirmed by PRNT. None of these individuals had expressed any symptoms in a questionnaire survey.

34. Beckett et al. (2005) performed an index case cluster study (35). Fifty three index cases were identified and family members and neighbors of the index cases were recruited. Of a total of 785 recruited individuals, there were eight asymptomatic and nine symptomatic dengue infections, yielding an inapparent rate of 47%. Symptomatology was defined as fever or any dengue-related symptoms.

**Multi-centric study - DENFRAME**

35. The DENFRAME FP6 European consortium performed a multi-centric index case household cluster analysis in Brazil, French Guyana, Cambodia and Viet Nam (36). There were 12 of 19 (63.2%) inapparent household member infections in the Latin American countries and 17 of 20 (85%) in the South East Asian countries. Symptomatology was based on fever and infection on IgM-positivity.

**Birth cohort – Maternal antibody study**

***Viet Nam***

36. Chau et al. (2009) performed a maternal antibody effect study on a birth cohort of 1,244 newborns followed every three months up to one year of age and then at 18 mths and two years (37). There were no infections up to three months of age and five infections from 3-6 months of age. Overall there were nine IgM-positive seroconversions and one case with a four-fold increase in IgG out of 819 individuals. Fever was reported in only one of these ten cases. Neutralizing antibodies were subsequently detected in all cases.

***Thailand***

37. Pengsaa et al. (2011) followed a birth cohort (N=55) and sampled the children at 3, 6, 9, 12, 18 and 24 months of age and then once a year from four to eight years of age (38). The parents were asked to report any febrile illness. During the first two years of life, there were eight dengue infections (by IgM seroconversion or a four-fold increase in PRNT) of which six were inapparent and two undifferentiated febrile illness (no “classical” dengue symptom). From four to eight years of age, there were an additional three symptomatic and 23 inapparent infections.

***Philippines***

38. Libraty et al. (2009) and Capeding et al. (2010) carried out a birth cohort follow-up for dengue infection in 4,441 infants to assess the effect of maternal antibody on outcome of infection (39, 40). Mothers were urged to bring their infants for clinical consultation in case of febrile illness. There were 40 confirmed symptomatic dengue cases. At the end of the year, a sample of 250 infants was randomly selected from those who had not presented with any clinical febrile illness. Twenty had seroconverted. This yields an inapparent rate of approximately 89%.

**Non-residents (Expats, Military & Travelers)**

In contrast to autochthonous populations living in areas endemic for dengue, expats, military personnel and travelers provide a special group of nominally naïve individuals who are pertinent to the question of importation of dengue into dengue-naïve regions. A few studies have addressed this by performing paired samples pre/post travel for serological survey coupled with a questionnaire of symptomatology.

39. Halstead et al. (1969) performed a seroconversion study using HAI, in white residents in Bangkok (41). There were five seroconversions of 395 individuals of whom four were ill (febrile illness).

40. Eight of twelve Japanese survey workers contracted symptomatic dengue (DENV1) infection (febrile illness); there were no inapparent infections (42). PRNT was used.

41. In American military personnel in Somalia, the majority of whom had never been to dengue-endemic countries previously, 44 of 494 individuals had IgM against DENV, of whom 33 had fever, four malaise (considered pauci-symptomatic) and seven were asymptomatic (43). MAC-ELISA, IgG indirect ELISA and viral isolation followed by IFA were performed.

42. A seroprevalence pre/post travel study performed on 104 Israeli travelers using IgG and IgM ELISA (PanBio assay) revealed five travelers were IgM-positive post travel and four reported illness. Three other travelers had a four-fold increase in IgG titer and did not report illness (44).

43. Cobelens et al. (2002) detected seroconversion in 13 of 447 Dutch travelers (45). Symptomatology defined as febrile illness plus one of headache, myalgia, arthralgia or rash. Three reported illness yielding an inapparent rate of 77%. Twelve were primary infections and one was secondary (based on IgG/IgM ratios using MAC-ELISA and IgG indirect ELISA).

44. Seet et al. (2005) observed a high symptomatic rate (24 of 27) in Chinese workers in Singapore; “few” were secondary infections. MAC-ELISA was used (46).

45. A serological diagnostic study was performed on 28 missionaries aged 16-69 years of age (21 provided serum samples for MAC-ELISA) returning from Haiti (CDC MMWR 2011). Seven of the 21 were seropositive, all of whom had consulted a clinician and reported dengue-like symptoms (47).

46. Baaten et al. (2011) studied 1207 travelers, of which 14 seroconverted but only five expressed any symptoms (MAC-ELISA and IgG indirect ELISA)(48).

47. Ratnam et al. (2012) studied Australian travelers (N=387) of whom four seroconverted (as detected by IgG/IgM, PanBio assay)(49). None expressed having any symptoms.

**References**

1. Barnes WJ, Rosen L. Fatal hemorrhagic disease and shock associated with primary dengue infection on a Pacific island. *Am J Trop Med Hyg* (1974) **23**: 495-506.
2. McBride WJ, Mullner H, LaBrooy JT, Wronski I. The 1993 dengue 2 epidemic in Charters Towers, North Queensland: clinical features and public health impact. *Epidemiol Infect* (1998) **121**: 151-6.
3. Guzmán MG, Kouri GP, Bravo J, Soler M, Vazquez S, Morier L. Dengue hemorrhagic fever in Cuba, 1981: a retrospective seroepidemiologic study. *Am J Trop Med Hyg* (1990) **42**: 179-84.
4. Kouri GP, Guzmán MG, Bravo JR, Triana C. Dengue haemorrhagic fever/dengue shock syndrome: lessons from the Cuban epidemic, 1981. Bull World Health Organ. 1989;67(4):375-80.
5. Guzmán MG, Kouri G, Valdes L, Bravo J, Alvarez M, Vazques S, Delgado I, Halstead SB. Epidemiologic studies on Dengue in Santiago de Cuba, 1997. *Am J Epidemiol* (2000) **152**: 793-9.
6. Likosky WH, Calisher CH, Michelson AL, Correa-Coronas R, Henderson BE, Feldman RA. An epidemiologic study of dengue type 2 in Puerto Rico, 1969. *Am J Epidemiol* (1973) **97**: 264-75.
7. Rodriguez-Figueroa L, Rigau-Perez JG, Suarez EL, Reiter P. Risk factors for dengue infection during an outbreak in Yanes, Puerto Rico in 1991. *Am J Trop Med Hyg* (1995) **52**: 496-502.
8. Lyerla R, Rigau-Pérez JG, Vorndam AV, Reiter P, George AM, Potter IM, Gubler DJ. A dengue outbreak among camp participants in a Caribbean island, 1995. *J Travel Med* (2000) **7**: 59-63.
9. Vasconcelos PF, Lima JW, da Rosa AP, Timbó MJ, da Rosa ES, Lima HR, Rodrigues SG, da Rosa JF. Dengue epidemic in Fortaleza, Ceará: randomized seroepidemiologic survey. *Rev Saude Publica* (1998) **32**: 447-54.
10. Rodrigues EM, Dal-Fabbro AL, Salomao R, Ferreira IB, Rocco IM, Fonseca BA. Epidemiology of dengue infection in Ribeirão Preto, SP, Brazil. *Rev Saude Publica* (2002) **36**: 160-5.
11. Méndez F, Barreto M, Arias JF, Rengifo G, Muñoz J, Burbano ME, Parra B. Human and mosquito infections by dengue viruses during and after epidemics in a dengue-endemic region of Colombia. *Am J Trop Med Hyg* (2006) **74**: 678-83.
12. Yeh WT, Chen RF, Wang L, Liu JW, Shaio MF, Yang KD. Implications of previous subclinical dengue infection but not virus load in dengue hemorrhagic fever. *FEMS Immunol Med Microbiol* (2006) **48**: 84-90.
13. Yap G, Li C, Mutalib A, Lai YL, Ng LC. High rates of inapparent dengue in older adults in Singapore. *Am J Trop Med Hyg* (2013) **88**: 1065-9. doi: 10.4269/ajtmh.12-0150.
14. Waterman SH, Novak RJ, Sather GE, Bailey RE, Rios I, Gubler DJ. Dengue transmission in two Puerto Rican communities in 1982. *Am J Trop Med Hyg* (1985) **34**: 625-32.
15. Balmaseda A, Hammond SN, Tellez Y, Imhoff L, Rodriguez Y, Saborío SI, Mercado JC, Perez L, Videa E, Almanza E, Kuan G, Reyes M, Saenz L, Amador JJ, Harris E. High seroprevalence of antibodies against dengue virus in a prospective study of schoolchildren in Managua, Nicaragua. *Trop Med Int Health* (2006) **11**: 935-42.
16. Balmaseda A, Standish K, Mercado JC, Matute JC, Tellez Y, Saborío S, Hammond SN, Nuñez A, Avilés W, Henn MR, Holmes EC, Gordon A, Coloma J, Kuan G, Harris E. Trends in patterns of dengue transmission over 4 years in a pediatric cohort study in Nicaragua. *J Infect Dis* (2010) **201**: 5-14. doi: 10.1086/648592.
17. Reyes M, Mercado JC, Standish K, Matute JC, Ortega O, Moraga B, Avilés W, Henn MR, Balmaseda A, Kuan G, Harris E. Index cluster study of dengue virus infection in Nicaragua. *Am J Trop Med Hyg* (2010) **83**: 683-9. doi: 10.4269/ajtmh.2010.10-0023.
18. Gordon A, Kuan G, Mercado JC, Gresh L, Avilés W, Balmaseda A, Harris E. The Nicaraguan pediatric dengue cohort study: incidence of inapparent and symptomatic dengue virus infections, 2004-2010. *PLoS Negl Trop Dis* (2013) **7**: e2462. doi: 10.1371/journal.pntd.0002462.
19. Montoya M, Gresh L, Mercado JC, Williams KL, Vargas MJ, Gutierrez G, Kuan G, Gordon A, Balmaseda A, Harris E. Symptomatic versus inapparent outcome in repeat dengue virus infections is influenced by the time interval between infections and study year. *PLoS Negl Trop Dis* (2013) **7**: e2357. doi: 10.1371/journal.pntd.0002357.
20. Honório NA, Nogueira RM, Codeço CT, Carvalho MS, Cruz OG, Magalhães Mde A, de Araújo JM, de Araújo ES, Gomes MQ, Pinheiro LS, da Silva Pinel C, Lourenço-de-Oliveira R. Spatial evaluation and modeling of Dengue seroprevalence and vector density in Rio de Janeiro, Brazil. *PLoS Negl Trop Dis* (2009) **3**: e545. doi: 10.1371/journal.pntd.0000545.
21. Morrison AC, Minnick SL, Rocha C, Forshey BM, Stoddard ST, Getis A, Focks DA, Russell KL, Olson JG, Blair PJ, Watts DM, Sihuincha M, Scott TW, Kochel TJ. Epidemiology of dengue virus in Iquitos, Peru 1999 to 2005: interepidemic and epidemic patterns of transmission. *PLoS Negl Trop Dis* (2010) **4**: e670. doi: 10.1371/journal.pntd.0000670.
22. Olkowski S, Forshey BM, Morrison AC, Rocha C, Vilcarromero S, Halsey ES, Kochel TJ, Scott TW, Stoddard ST. Reduced risk of disease during postsecondary dengue virus infections*. J Infect Dis* (2013) **208**: 1026-33.
23. Burke DS, Nisalak A, Johnson DE, Scott RM. A prospective study of dengue infections in Bangkok. *Am J Trop Med Hyg* (1988) **38**: 172-80.
24. Endy TP, Chunsuttiwat S, Nisalak A, Libraty DH, Green S, Rothman AL, Vaughn DW, Ennis FA. Epidemiology of inapparent and symptomatic acute dengue virus infection: a prospective study of primary school children in Kamphaeng Phet, Thailand. *Am J Epidemiol* (2002) **156**: 40-51.
25. Anderson KB, Gibbons RV, Thomas SJ, Rothman AL, Nisalak A, Berkelman RL, Libraty DH, Endy TP. Preexisting Japanese encephalitis virus neutralizing antibodies and increased symptomatic dengue illness in a school-based cohort in Thailand. *PLoS Negl Trop Dis* (2011) **5**: e1311. doi: 10.1371/journal.pntd.0001311.
26. Endy TP, Anderson KB, Nisalak A, Yoon IK, Green S, Rothman AL, Thomas SJ, Jarman RG, Libraty DH, Gibbons RV. Determinants of inapparent and symptomatic dengue infection in a prospective study of primary school children in Kamphaeng Phet, Thailand. *PLoS Negl Trop Dis* (2011) **5**: e975. doi: 10.1371/journal.pntd.0000975
27. Mammen MP, Pimgate C, Koenraadt CJ, Rothman AL, Aldstadt J, Nisalak A, Jarman RG, Jones JW, Srikiatkhachorn A, Ypil-Butac CA, Getis A, Thammapalo S, Morrison AC, Libraty DH, Green S, Scott TW. Spatial and temporal clustering of dengue virus transmission in Thai villages. *PLoS Med* (2008) **5**: e205. doi: 10.1371/journal.pmed.0050205.
28. Yoon IK, Rothman AL, Tannitisupawong D, Srikiatkhachorn A, Jarman RG, Aldstadt J, Nisalak A, Mammen MP Jr, Thammapalo S, Green S, Libraty DH, Gibbons RV, Getis A, Endy T, Jones JW, Koenraadt CJ, Morrison AC, Fansiri T, Pimgate C, Scott TW. Underrecognized mildly symptomatic viremic dengue virus infections in rural Thai schools and villages. *J Infect Dis* (2012) **206**: 389-98. doi: 10.1093/infdis/jis357.
29. Yoon IK, Srikiatkhachorn A, Hermann L, Buddhari D, Scott TW, Jarman RG, Aldstadt J, Nisalak A, Thammapalo S, Bhoomiboonchoo P, Mammen MP, Green S, Gibbons RV, Endy TP, Rothman AL. Characteristics of mild dengue virus infection in thai children. *Am J Trop Med Hyg* (2013) **89**: 1081-7. doi: 10.4269/ajtmh.13-0424.
30. Van Benthem BH, Vanwambeke SO, Khantikul N, Burghoorn-Maas C, Panart K, Oskam L, Lambin EF, Somboon P. Spatial patterns of and risk factors for seropositivity for dengue infection. *Am J Trop Med Hyg* (2005) **72**: 201-8.
31. Vanwambeke SO, van Benthem BH, Khantikul N, Burghoorn-Maas C, Panart K, Oskam L, Lambin EF, Somboon P. Multi-level analyses of spatial and temporal determinants for dengue infection. *Int J Health Geogr* (2006) **5**:5.
32. Anderson KB, Gibbons RV, Cummings DA, Nisalak A, Green S, Libraty DH, Jarman RG, Srikiatkhachorn A, Mammen MP, Darunee B, Yoon IK, Endy TP. A Shorter Time Interval Between First and Second Dengue Infections Is Associated With Protection From Clinical Illness in a School-based Cohort in Thailand. *J Infect Dis* (2014) **209**: 360-8. doi: 10.1093/infdis/jit436.
33. Tien NT, Luxemburger C, Toan NT, Pollissard-Gadroy L, Huong VT, Van Be P, Rang NN, Wartel TA, Lang J. A prospective cohort study of dengue infection in schoolchildren in Long Xuyen, Viet Nam. *Trans R Soc Trop Med Hyg* (2010) **104**: 592-600. doi: 10.1016/j.trstmh.2010.06.003.
34. Porter KR, Beckett CG, Kosasih H, Tan RI, Alisjahbana B, Rudiman PI, Widjaja S, Listiyaningsih E, Ma'Roef CN, McArdle JL, Parwati I, Sudjana P, Jusuf H, Yuwono D, Wuryadi S. Epidemiology of dengue and dengue hemorrhagic fever in a cohort of adults living in Bandung, West Java, Indonesia. *Am J Trop Med Hyg* (2005) **72**: 60-6.
35. Beckett CG, Kosasih H, Faisal I, Nurhayati, Tan R, Widjaja S, Listiyaningsih E, Ma'roef C, Wuryadi S, Bangs MJ, Samsi TK, Yuwono D, Hayes CG, Porter KR. Early detection of dengue infections using cluster sampling around index cases. *Am J Trop Med Hyg* (2005) **72**: 777-82.
36. Dussart P, Baril L, Petit L, Beniguel L, Quang LC, Ly S, Azevedo Rdo S, Meynard JB, Vong S, Chartier L, Diop A, Sivuth O, Duong V, Thang CM, Jacobs M, Sakuntabhai A, Nunes MR, Huong VT, Buchy P, Vasconcelos PF. Clinical and virological study of dengue cases and the members of their households: the multinational DENFRAME Project. *PLoS Negl Trop Dis* (2012) **6**: e1482. doi: 10.1371/journal.pntd.0001482.
37. Chau TN, Hieu NT, Anders KL, Wolbers M, Lien le B, Hieu LT, Hien TT, Hung NT, Farrar J, Whitehead S, Simmons CP. Dengue virus infections and maternal antibody decay in a prospective birth cohort study of Vietnamese infants. *J Infect Dis* (2009) **200**: 1893-900. doi: 10.1086/648407.
38. Pengsaa K, Limkittikul K, Yoksan S, Wisetsing P, Sabchareon A. Dengue antibody in Thai children from maternally transferred antibody to acquired infection. *Pediatr Infect Dis J* (2011) **30**: 897-900. doi: 10.1097/INF.0b013e31821f07f6.
39. Libraty DH, Acosta LP, Tallo V, Segubre-Mercado E, Bautista A, Potts JA, Jarman RG, Yoon IK, Gibbons RV, Brion JD, Capeding RZ. A prospective nested case-control study of Dengue in infants: rethinking and refining the antibody-dependent enhancement dengue hemorrhagic fever model. *PLoS Med* (2009) **6**: e1000171. doi: 10.1371/journal.pmed.1000171.
40. Capeding RZ, Brion JD, Caponpon MM, Gibbons RV, Jarman RG, Yoon IK, Libraty DH. The incidence, characteristics, and presentation of dengue virus infections during infancy. *Am J Trop Med Hyg* (2010) **82**: 330-6. doi: 10.4269/ajtmh.2010.09-0542.
41. Halstead SB, Udomsakdi S, Singharaj P, Nisalak A. Dengue chikungunya virus infection in man in Thailand, 1962-1964. 3. Clinical, epidemiologic, and virologic observations on disease in non-indigenous white persons. *Am J Trop Med Hyg* (1969) **18**: 984-96.
42. Fukunaga T, Okuno Y, Tadano M, Fukai K. A retrospective serological study of Japanese who contracted dengue fever in Thailand. *Biken J* (1983) **26**: 67-74.
43. Sharp TW, Wallace MR, Hayes CG, Sanchez JL, DeFraites RF, Arthur RR, Thornton SA, Batchelor RA, Rozmajzl PJ, Hanson RK, Wu SJ, Iriye C, Burans JP. Dengue fever in U.S. troops during Operation Restore Hope, Somalia, 1992-1993. *Am J Trop Med Hyg* (1995) **53**: 89-94.
44. Potasman I, Srugo I, Schwartz E. Dengue seroconversion among Israeli travelers to tropical countries. *Emerg Infect Dis* (1999) **5**: 824-7.
45. Cobelens FG, Groen J, Osterhaus AD, Leentvaar-Kuipers A, Wertheim-van Dillen PM, Kager PA. Incidence and risk factors of probable dengue virus infection among Dutch travellers to Asia. *Trop Med Int Health* (2002) **7**: 331-8.
46. Seet RC, Ooi EE, Wong HB, Paton NI. An outbreak of primary dengue infection among migrant Chinese workers in Singapore characterized by prominent gastrointestinal symptoms and a high proportion of symptomatic cases. *J Clin Virol* (2005) **33**: 336-40.
47. CDC. Dengue virus infection among travellers returning from Haiti – Georgia and Nebraska, October 2010. *MMWR* (2011) **60**: 914-7.
48. Baaten GG, Sonder GJ, Zaaijer HL, van Gool T, Kint JA, van den Hoek A. Travel-related dengue virus infection, The Netherlands, 2006-2007. *Emerg Infect Dis* (2011) 17: 821-8. doi: 10.3201/eid1705.101125.
49. Ratnam I, Black J, Leder K, Biggs BA, Matchett E, Padiglione A, Woolley I, Panagiotidis T, Gherardin T, Pollissard L, Demont C, Luxemburger C, Torresi J. Incidence and seroprevalence of dengue virus infections in Australian travellers to Asia. *Eur J Clin Microbiol Infect Dis* (2012) **31**: 1203-10. doi: 10.1007/s10096-011-1429-1.
